# Exploring Potential for Repurposing Antiretroviral Drugs Etravirine and Efavirenz in Prostate and Bladder Cancer

**DOI:** 10.3390/ph18091404

**Published:** 2025-09-18

**Authors:** Mariana Pereira, Nuno Vale

**Affiliations:** 1 PerMed Research Group, RISE-Health, Faculty of Medicine, University of Porto, Alameda Professor Hernâni Monteiro, 4200-319 Porto, Portugal; mariana.m.pereira2097@gmail.com; 2 ICBAS—School of Medicine and Biomedical Sciences, University of Porto, Rua de Jorge Viterbo Ferreira, 228, 4050-313 Porto, Portugal; 3 RISE-Health, Department of Community Medicine, Health Information and Decision (MEDCIDS), Faculty of Medicine, University of Porto, Rua Doutor Plácido da Costa, 4200-450 Porto, Portugal; 4 Laboratory of Personalized Medicine, Department of Community Medicine, Health Information and Decision (MEDCIDS), Faculty of Medicine, University of Porto, Rua Doutor Plácido da Costa, 4200-450 Porto, Portugal

**Keywords:** prostate cancer, bladder cancer, etravirine, efavirenz, drug repurposing, drug combination

## Abstract

**Background/Objectives:** Prostate and bladder cancers are significant global health challenges with increasing incidence and limited treatment options in advanced stages. Drug repurposing offers a cost-effective strategy to accelerate the development of new anticancer therapies. This study investigated the antitumor activity of the non-nucleoside reverse transcriptase inhibitors efavirenz (EFV) and etravirine (ETV) in prostate and bladder cancer models. **Methods:** PC-3 prostate cancer and UM-UC-5 bladder cancer cell lines were treated with EFV, ETV, or their combination. Cell viability was assessed at 24, 48, and 72 h to evaluate time and concentration-dependent effects. Wound-healing assays were used to measure cell migration, and clonogenic assays assessed long-term proliferative capacity. **Results:** Both EFV and ETV decreased cell viability in a time and dose-dependent manner. ETV showed greater potency in PC-3 cells, while EFV demonstrated more consistent effects in UM-UC-5 cells. Combination treatment enhanced cytotoxicity, particularly at 48 and 72 h, suggesting potential synergy. Wound-healing assays indicated impaired migration in UM-UC-5 cells treated with ETV or the EFV + ETV combination. Clonogenic assays confirmed reduced long-term proliferation in both cell lines following treatment. **Conclusions**: EFV and ETV exhibit selective anticancer activity in prostate and bladder cancer cells, with enhanced effects when combined. These findings support their potential as repurposed therapeutic agents and warrant further preclinical evaluation for prostate and bladder cancer therapy.

## 1. Introduction

As of 2022 according to the Global Cancer Observatory, prostate cancer has more than 1.4 million cases and almost 400,000 deaths worldwide, being the cancer with the fourth highest incidence and eighth highest mortality rate [1]. The incidence of advanced metastatic prostate cancer has been increasing 5% per year in the later years and in this stage, this disease remains lethal [2]. Although some forms of treatments already exist for both castration-sensitive, via androgen deprivation therapy and anti-cancer drugs like docetaxel or abiraterone [3], and resistant metastatic prostate cancer, through radioligand therapy and drugs like olaparib and rucaparib [4], future research in this area is still imperative.

Bladder cancer, as of 2022 and according to GLOBOCAN, has the 9th highest worldwide cancer incidence, with over 600,000 new cases, and is particularly incident in men, where it occupies the 6th place [5]. Bladder cancer is typically very heterogeneous, with several histological and clinical types, with various genetic alterations, which makes it a difficult subject for treatment [6]. This makes bladder cancer therapy research a continuous study in medicine.

One method from which new cancer therapies could be derived is drug repurposing, also known as drug repositioning or drug reprofiling, an emerging strategy focused on finding new therapeutic applications for existing drugs rather than starting from scratch. This approach offers several advantages, including cost-effectiveness and faster clinical translation, as repurposed drugs have already undergone safety assessments in humans [7]. This method is garnering increasing interest in several areas, including cancer. One of the main groups of drugs that are studied for cancer repurposing is antivirals. In recent years, several literature reviews have been made on this subject, and such repurposing includes ritonavir, nelfinavir, acyclovir, ribavirin, cidofovir, favipiravir, lopinavir, and efavirenz, in various cancer types such as lymphoma, glioblastomas, cervical, breast, pancreatic, and prostate cancers [8,9,10,11,12,13].

**Figure 1:**
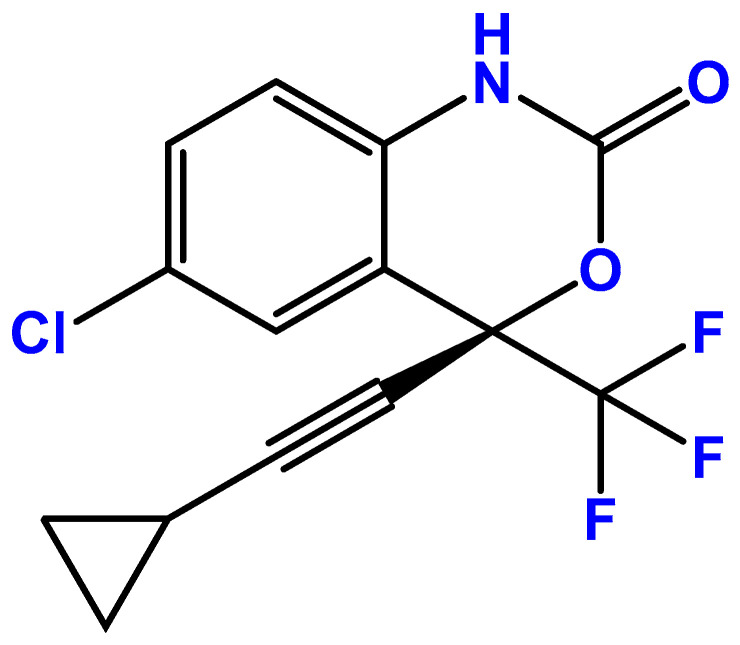
Chemical structures of efavirenz (EFV). Developed with ChemBioDraw^®^ Ultra version 13.0., a chemical drawing software (PerkinElmer Inc., Waltham, MA, USA).

Efavirenz, originally developed by DuPont Pharmaceuticals, is a first-generation benzoxazinone non-nucleoside reverse transcriptase inhibitor (NNRTI) that is used to treat human immunodeficiency virus (HIV) type 1 infection or stop HIV from spreading (Figure 1). It is marketed under the trade names Sustiva^®^ and Stocrin^®^. On 7 June 1998, the Food and Drug Administration (FDA) approved efavirenz for the treatment of HIV infection at a dosage of 600 mg taken once daily orally. The European Union approved the medication in 1999 [14]. Later research found that EFV’s recommended dosage may be lowered to 400 mg while still providing comparable viral suppression, which would lower costs and increase the drug’s safety [15]. EFV is dependent on intracellular conversion to the active triphosphorylated form to suppress the action of viral RNA-directed DNA polymerase, also known as reverse transcriptase [16]. This drug has been proven to have potential in repurposing for several diseases, such as Prion disease [17] and cancers such as lung cancer and leukemia [18,19], which is why it was selected for our study.

**Figure 2:**
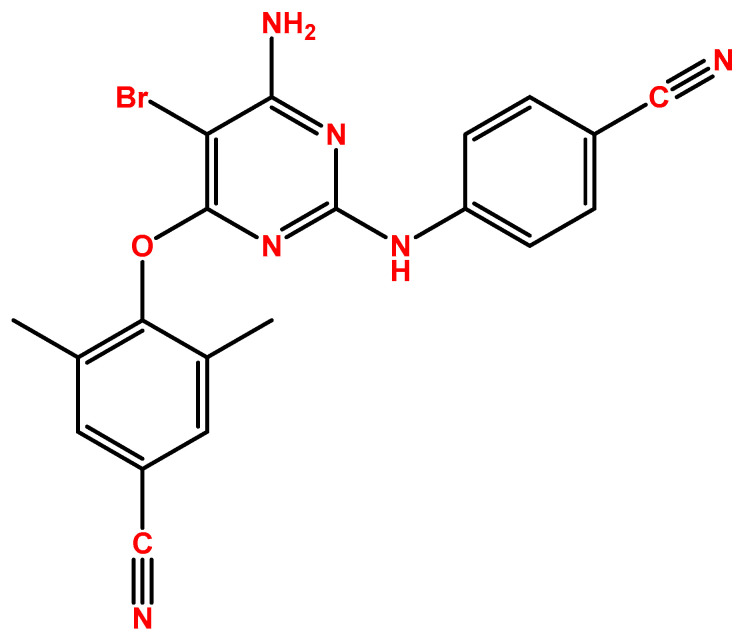
Chemical structures of etravirine (ETV). Developed with ChemBioDraw^®^ Ultra version 13.0., a chemical drawing software (PerkinElmer Inc., Waltham, MA, USA).

Etravirine (ETV), marketed as Intelence^®^, represents a second-generation NNRTI introduced in 2007 in the United States (Figure 2). It is primarily utilized in combination therapies for patients with prior treatment experience [20]. ETV exerts its therapeutic effects by inhibiting both RNA- and DNA-dependent polymerase activities. This inhibition occurs allosterically, meaning it binds in a specific pocket adjacent to the catalytic site of reverse transcriptase, thereby disrupting the enzyme’s function. Consequently, the synthesis of viral complementary DNA (cDNA) is impeded. Beyond its direct impact on polymerase activity, ETV also influences the post-integration stages of the HIV replication cycle. It appears to enhance the processing of precursor proteins, such as gag and gag-pol, within HIV-1 transfected cells. This modulation ultimately reduces the production of viable viral particles, contributing to its efficacy in controlling HIV infection [21]. This investigation group has shown ETV to have potential for repurposing, by demonstrating its effects in decreasing the cell viability of bladder cancer cells [22].

This study aimed to evaluate the potential repurposing of the antiretroviral drugs EFV and ETV as anti-cancer agents against two different cancer types: prostate cancer (PC-3 cell line) and bladder cancer (UM-UC-5 cell line). Among NNRTIs, EFV and ETV were selected for this study based on their superior anti-cancer profiles compared to other drugs in the class. Comparative in vitro studies have demonstrated that EFV exhibits a markedly higher cytotoxic effect against cancer cells than other NNRTIs, with the 50% effective cytotoxic concentration (EC50) of EFV being approximately eight times lower than that of other NNRTIs, like NVP, in pancreatic cancer cell lines (EFV: 31.5 μmol/L vs. NVP: 239 μmol/L) [23]. This suggests that EFV is substantially more potent in reducing cancer cell viability. Mechanistically, EFV’s higher affinity for LINE-1 encoded reverse transcriptase makes it a more powerful blocker of RT function in cancer cells, contributing to its pronounced anti-proliferative and differentiation-inducing effects [24]. Meanwhile, ETV was chosen due to its demonstrated efficacy in reducing cell viability in bladder cancer models by our group, with IC_50_ values in the low micromolar range and additional potential mechanisms such as inhibition of casein kinase 1ε and AGR2, both implicated in cancer cell survival and proliferation [22]. These properties, combined with favorable pharmacological profiles and prior evidence of anti-cancer activity, provided a strong rationale for prioritizing EFV and ETV over other NNRTIs for evaluation in our study.

The primary objective was to assess the cytotoxic effects of EFV and ETV, individually and in combination, on cancer cell viability across a range of concentrations and exposure times. To further characterize the anti-cancer potential of these compounds, we examined their impact on cell migration using wound healing assays and on long-term proliferative capacity using clonogenic assays. Finally, to evaluate their biosafety, the effects of EFV, ETV, and their combination were tested in non-cancerous MRC-5 human fibroblasts to assess selective cytotoxicity.

## 2. Results

### 2.1. Viability—PC-3

**Figure 3:**
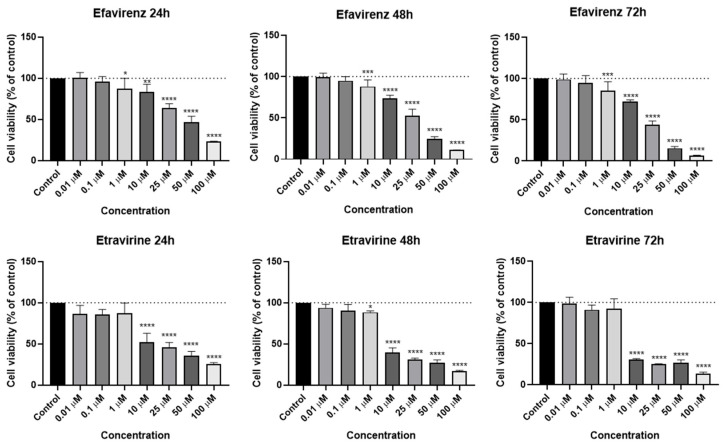
PC-3 cell viability was measured after 24, 48, and 72 h of treatment with increasing concentrations (0.01–100 µM) of EFV and ETV. DMSO at 0.01% served as the vehicle control. Viability was determined using the MTT assay, with data presented as mean ± SEM (*n* = 3). Statistical significance compared to the vehicle control is indicated as follows: * *p* < 0.05; ** *p* < 0.01; *** *p* < 0.001; **** *p* < 0.0001.

**Figure 4:**
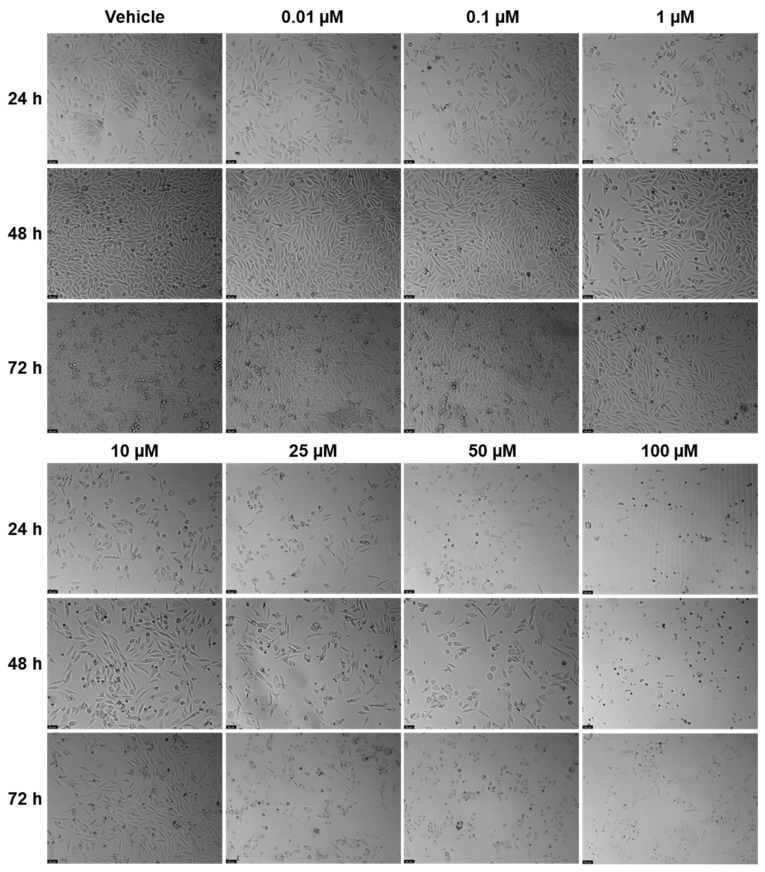
Morphological changes in PC-3 cells were examined after 24, 48, and 72 h of treatment with escalating concentrations of EFV (0.01–100 µM; *n* = 3). Control cells were treated with 0.01% DMSO as the vehicle. The scale bar represents 50 µm.

**Figure 5:**
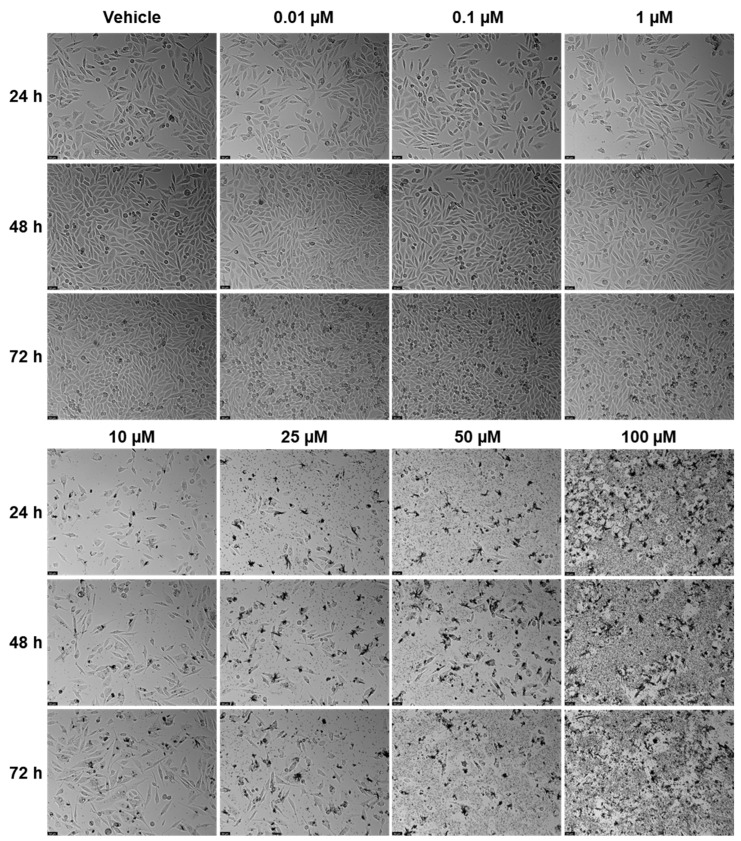
Morphological changes in PC-3 cells were examined after 24, 48, and 72 h of treatment with escalating concentrations of ETV (0.01–100 µM; *n* = 3). Control cells were treated with 0.01% DMSO as the vehicle. The scale bar represents 50 µm.

EFV and ETV were tested in PC-3 metastatic prostate cancer cells at concentrations of 0.01, 0.1, 1, 10, 25, 50, and 100 µM for three time periods (24 h, 48 h, and 72 h). Figure 3 shows the outcomes of cell viability testing, and Figure 4 and Figure 5 show the morphological examinations. EFV presents a clear concentration-dependent effect on prostate cancer cells, with a statistically significant decrease in cell viability at all time points from a low concentration of 1 µM. Although less marked, there is also a time-dependent effect, with longer times having a more prominent decrease in cell viability for the same concentration. This effect is more accentuated for higher concentrations (25 µM and higher). In the cell images, it can be seen a decrease in cellular density, as well as a transition of cell morphology from their usual elongated form to a rounder shape with quite a few burst cells as the concentrations become higher (Figure 4). ETV consistently demonstrates a more potent inhibitory effect on PC-3 cell viability compared to EFV across all assessed time points. When comparing equivalent concentrations, ETV induces a greater decrease in cell survival than EFV, with 10 µM of ETV being sufficient to reduce viability by at least 50% at every time point measured. A plateau effect becomes evident at later stages, particularly beyond 48 h, where increasing the concentration from 10 µM to 100 µM results in only marginal additional reductions in viability. Similarly, the differences in cell viability between the 48 h and 72 h marks are minimal, suggesting that the maximum cytotoxic effect of ETV is reached relatively early and that further exposure time or higher doses do not significantly enhance its efficacy.

### 2.2. Viability—UM-UC-5

**Figure 6:**
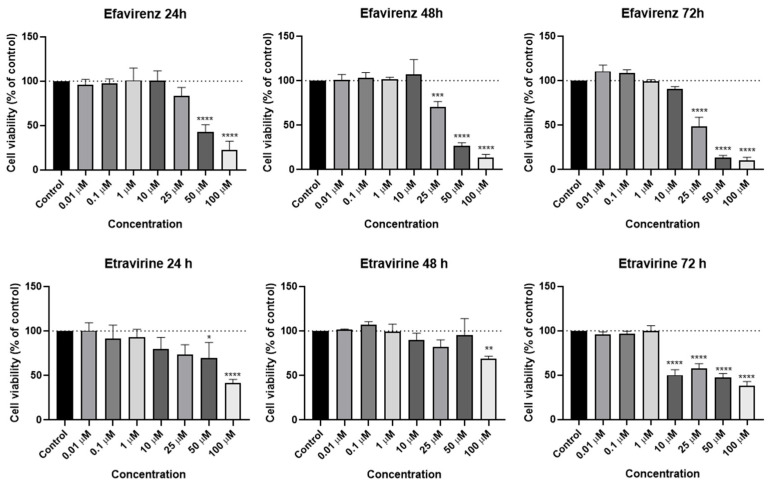
UM-UC-5 cell viability was measured after 24, 48, and 72 h of treatment with increasing concentrations (0.01–100 µM) of EFV and ETV. DMSO at 0.01% served as the vehicle control. Viability was determined using the MTT assay, with data presented as mean ± SEM (*n* = 3). Statistical significance compared to the vehicle control is indicated as follows: * *p* < 0.05; ** *p* < 0.01; *** *p* < 0.001; **** *p* < 0.0001.

**Figure 7:**
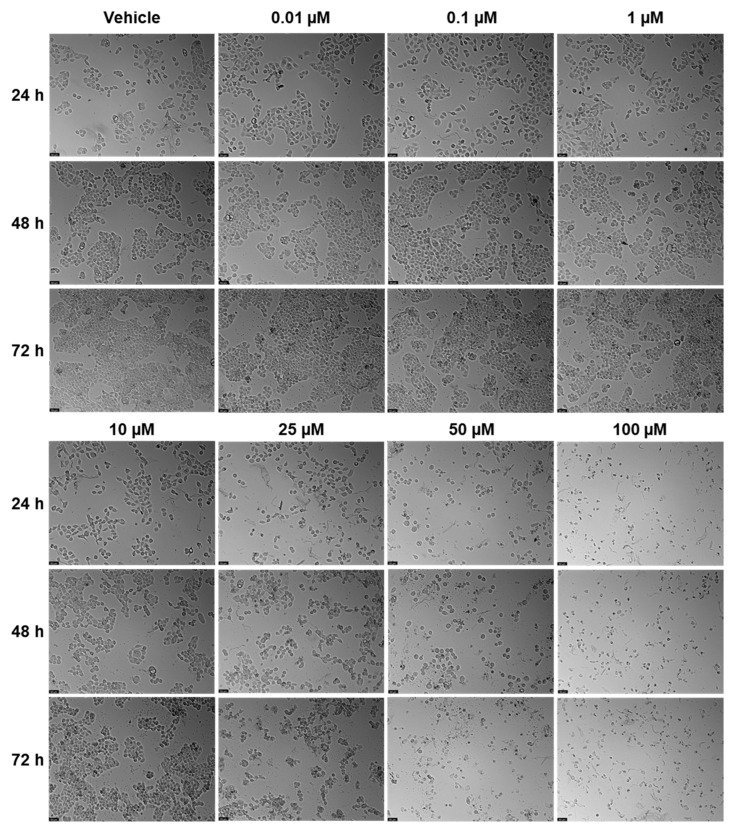
Morphological changes in UM-UC-5 cells were examined after 24, 48, and 72 h of treatment with escalating concentrations of EFV (0.01–100 µM; *n* = 3). Control cells were treated with 0.01% DMSO as the vehicle. The scale bar represents 50 µm.

**Figure 8:**
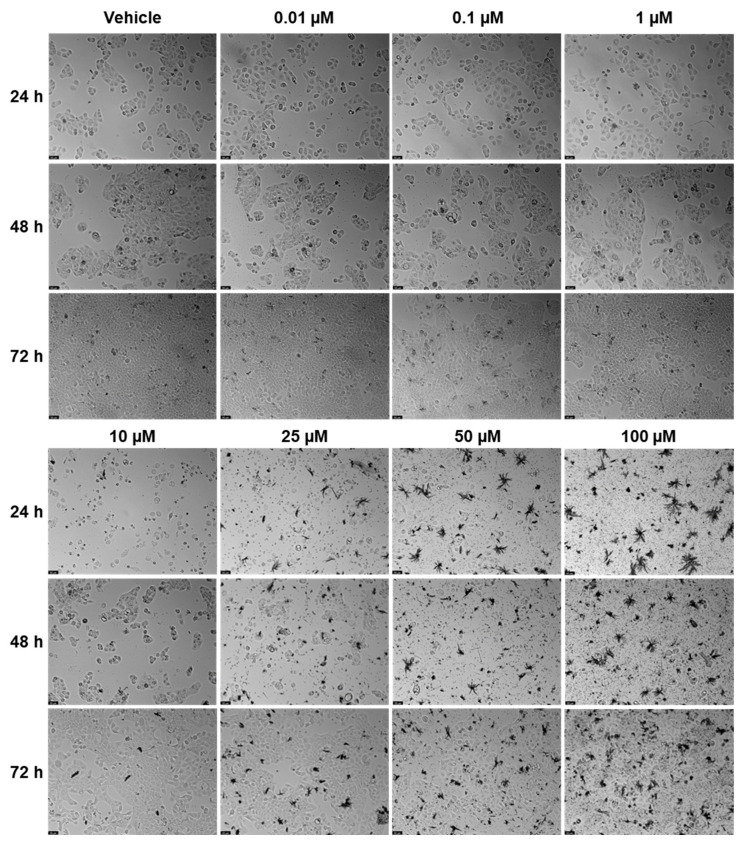
Morphological changes in UM-UC-5 cells were examined after 24, 48, and 72 h of treatment with escalating concentrations of ETV (0.01–100 µM; *n* = 3). Control cells were treated with 0.01% DMSO as the vehicle. The scale bar represents 50 µm.

EFV and ETV were tested in UM-UC-5 bladder cancer cells at concentrations of 0.01, 0.1, 1, 10, 25, 50, and 100 µM for three time periods (24 h, 48 h, and 72 h), and the results can be seen in Figure 6, Figure 7 and Figure 8. EFV exhibits a concentration- and time-dependent decrease in cell viability (Figure 6). At 24 h, a significant reduction is seen at higher concentrations, with cell viability dropping to approximately 50% or below at 50 and 100 µM. At 48 and 72 h, the cytotoxic effect of EFV becomes more pronounced, with lower concentrations (10 µM) already causing substantial reductions in viability. By 72 h, even intermediate concentrations (10–25 µM) significantly reduce cell survival, suggesting that longer exposure enhances EFV’s potency. ETV also reduces UM-UC-5 cell viability, though its effect is somewhat less concentration-dependent at earlier time points compared to EFV. At 24 and 48 h, there is a reduction in cell viability, but only at higher concentrations. It is at 72 h that the cytotoxic effects become more evident, where concentrations of 10 µM and above significantly decrease viability. However, this reduction is similar from 10 to 100 µM, indicating that, as happens in PC-3, there is a plateau at a certain concentration.

In controls and at lower drug concentrations (0.01–1 µM), cells maintained their typical epithelial morphology, forming dense monolayers with cell–cell junctions throughout the 72 h period. At higher EFV concentrations (10–100 µM), morphological alterations became evident. By 24 h, cells treated with 50 and 100 µM showed signs of cytotoxic stress, including cell rounding, loss of adherence, and reduced monolayer integrity. These effects intensified at 48 h and 72 h, with widespread detachment and cellular debris indicating extensive cell death. At intermediate concentrations (10–25 µM), moderate changes such as partial monolayer disruption and reduced cell density were apparent by 72 h (Figure 7). Meanwhile, for ETV, at concentrations of 10 µM and above, cells began showing signs of stress, including slight rounding and reduced density, particularly at 48 h and 72 h. By 72 h, higher doses (10–100 µM) caused pronounced morphological disruption, with rounded and shrunken cells, detachment from the surface, and scattered debris. Notably, the extent of these changes did not markedly increase between 10 and 100 µM and was never as pronounced as with EFV, suggesting, as previously mentioned, a plateau effect in morphological damage at higher doses. Increased debris in ETV can also be associated with drug precipitates (Figure 8).

The calculated IC_50_ for both drugs at all time points and cell lines can be seen in Table 1. In PC-3 cells, both compounds exhibited a time-dependent decrease in IC_50_ values, indicating increased potency with longer exposure. ETV consistently showed lower IC_50_ values than EFV at all time points, suggesting greater efficacy in reducing PC-3 cell viability.

In UM-UC-5 cells, EFV also demonstrated a time-dependent reduction in IC_50_ values (from 48.32 µM at 24 h to 24.59 µM at 72 h). ETV, however, displayed a less consistent pattern in this cell line, with inconsistent values at 24 h and 48 h, and only decreasing to 33.51 µM at 72 h. These results suggest that while both compounds are effective in reducing cell viability over time, ETV is more potent in PC-3 cells, whereas EFV demonstrates comparatively greater and more consistent cytotoxicity in UM-UC-5 cells.

### 2.3. Combination Studies

**Figure 9:**
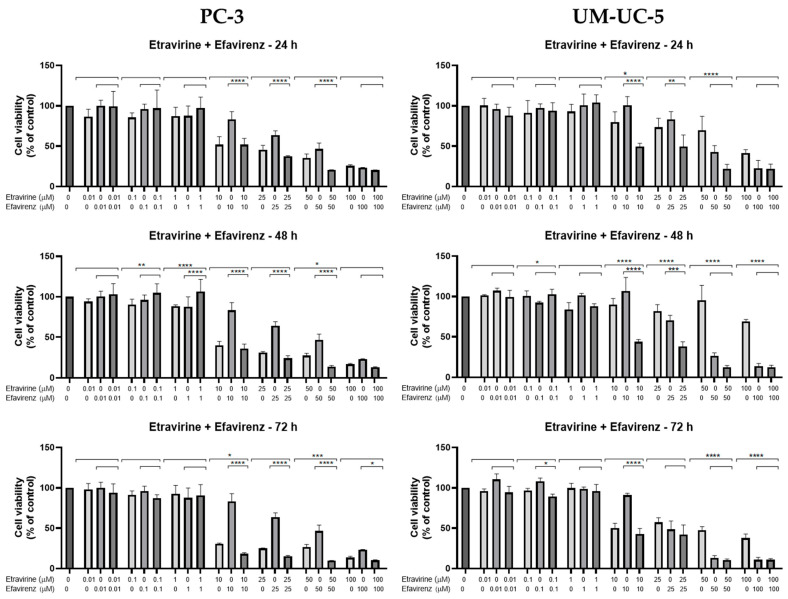
Cytotoxicity results for PC-3 (**left**) and UM-UC-5 (**right**) cells after 24, 48, and 72 h of treatment with individual drugs and their combination (EFV and ETV administered simultaneously) are presented. Negative control cells received 0.1% DMSO as the vehicle. Cell viability was assessed using the MTT assay, with data expressed as mean ± SEM (*n* = 3). Statistical significance compared to single-drug treatments is indicated as: * *p* < 0.05, ** *p* < 0.01, *** *p* < 0.001, and **** *p* < 0.0001.

**Figure 10:**
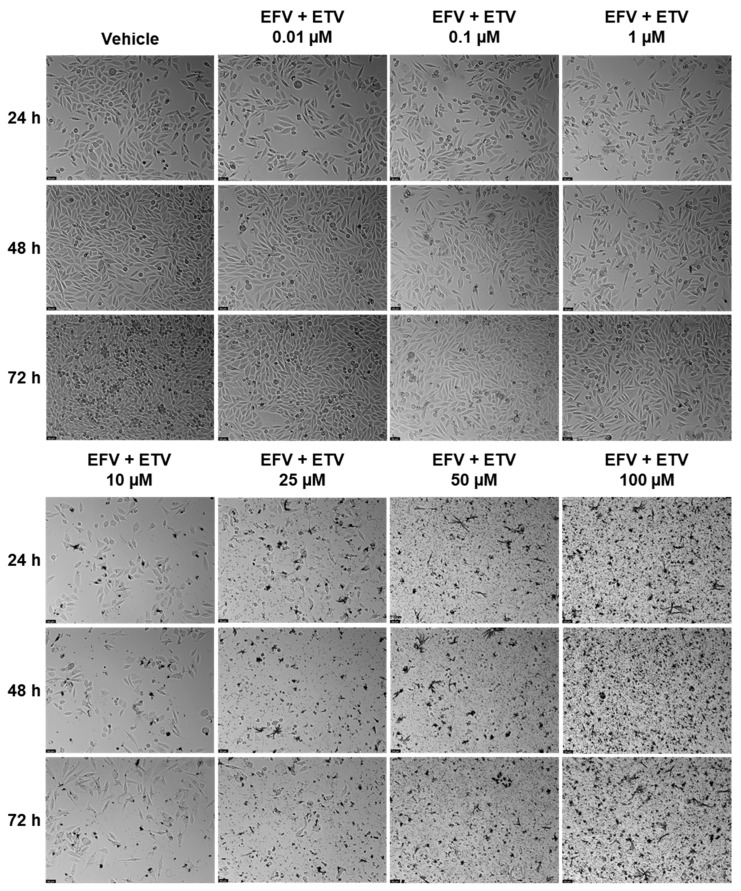
PC-3 cell morphology was evaluated after treatment with combined EFV and ETV at concentrations ranging from 0.01 to 100 µM for 24, 48, and 72 h (*n* = 3). Control cells were treated with 0.01% DMSO as the vehicle. The scale bar indicates 50 µm.

**Figure 11:**
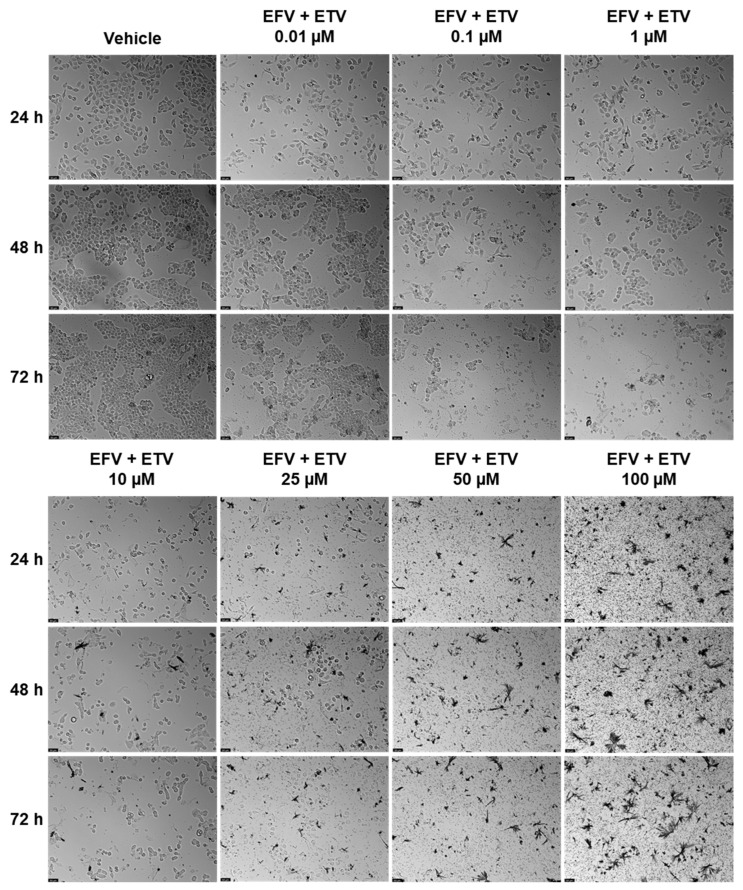
UM-UC-5 cell morphology was evaluated after treatment with combined EFV and ETV at concentrations ranging from 0.01 to 100 µM for 24, 48, and 72 h (*n* = 3). Control cells were treated with 0.01% DMSO as the vehicle. The scale bar indicates 50 µm.

Combination studies of EFV and ETV, at the same concentrations, were performed in both cell lines for all three time points, and the results of cell viability and morphology can be seen in Figure 9 and in Figure 10 and Figure 11, respectively.

In PC-3 cells, at 48 h, the combination of EFV and ETV at 50 µM showed significant improvements compared to both single agents, as indicated by the presence of asterisks for both EFV and ETV at that dose. In contrast, at 25 µM, although the combination was significantly better than EFV alone, it was not significantly better than ETV, indicating that the observed decrease might primarily reflect the effect of ETV rather than a true synergistic interaction. Similar patterns were observed at other time points, with clearer combination effects emerging at later stages (48 h and 72 h) (Figure 9). Figure 10 shows the decrease in cell density as well as the loss of the elongated nature of PC-3 cells with increased concentration combinations.

In UM-UC-5 cells, only at 48 h did combinations show synergy in comparison with single treatments, for both 10 and 25 µM, while at higher concentrations, the effect was almost all due to EFV (Figure 9). Figure 11 demonstrates the decrease in cell viability from the graph bars, with a clear decrease in the aggregation of cells when compared with the control and lower concentrations.

Overall, these results suggest that while both cell lines benefit from combination treatment, UM-UC-5 cells exhibit greater sensitivity and more consistent enhancement of cytotoxicity with EFV and ETV combined, particularly at later time points. This highlights a potential therapeutic advantage of combination therapy in bladder cancer cells.

### 2.4. Wound Healing Assays

The effect of EFV and ETV, alone or in combination, on cell motility was assessed using a wound healing assay, for concentrations of 10 and 25 µM for 24, 48, and 72 h. These concentrations were chosen since these were the concentrations at which, generally, a cytotoxic impact started in both cell lines and around some of the IC_50_ values calculated.

**Figure 12:**
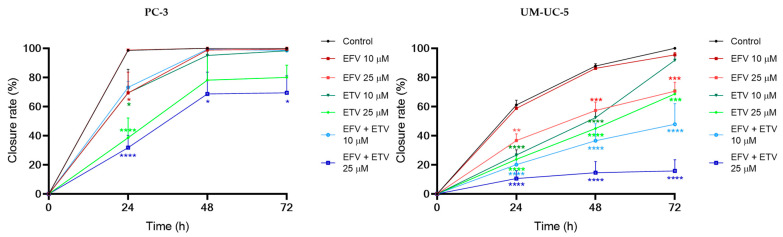
Statistical analysis of migration results derived from the images is presented as percentages relative to the control group, expressed as mean ± SD. Each experiment was conducted independently in quadruplicate (*n* = 3). Statistical significance compared to single-drug treatment is denoted as follows: * *p* < 0.05, ** *p* < 0.01, *** *p* < 0.001, and **** *p* < 0.0001.

**Figure 13:**
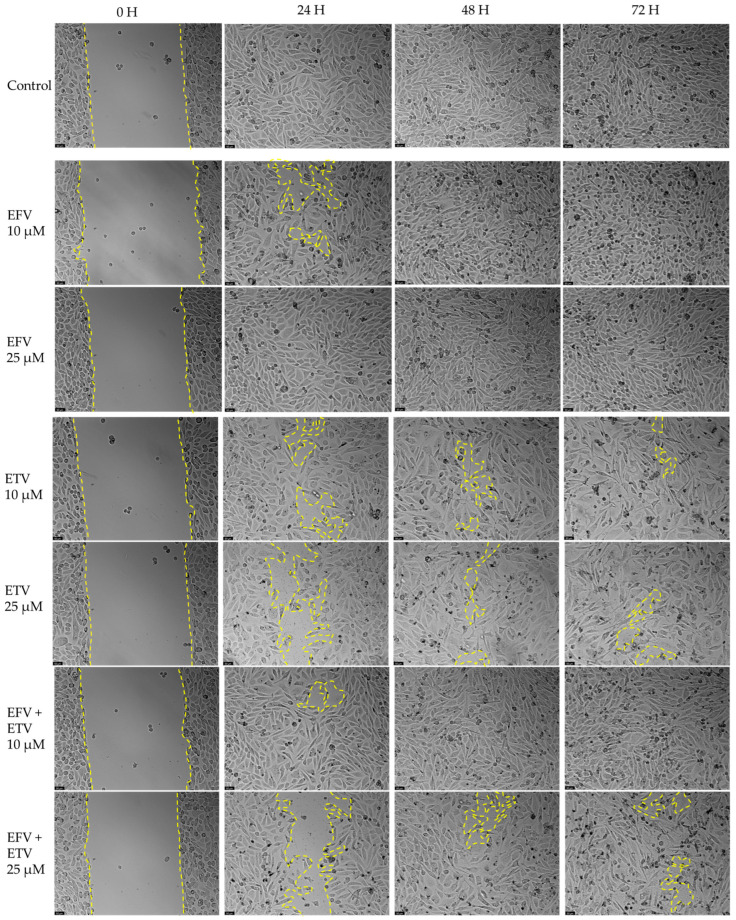
Representative images from in vitro wound healing assays in PC-3 prostate cancer cells, captured at 0, 24, 48, and 72 h following treatment with EFV, ETV, or their combination. The images reflect three independent experiments performed in quadruplicate. Dashed lines are included as visual guides to indicate cell migration trends and are not intended to represent precise measurements. The scale bar represents 50 μm.

**Figure 14:**
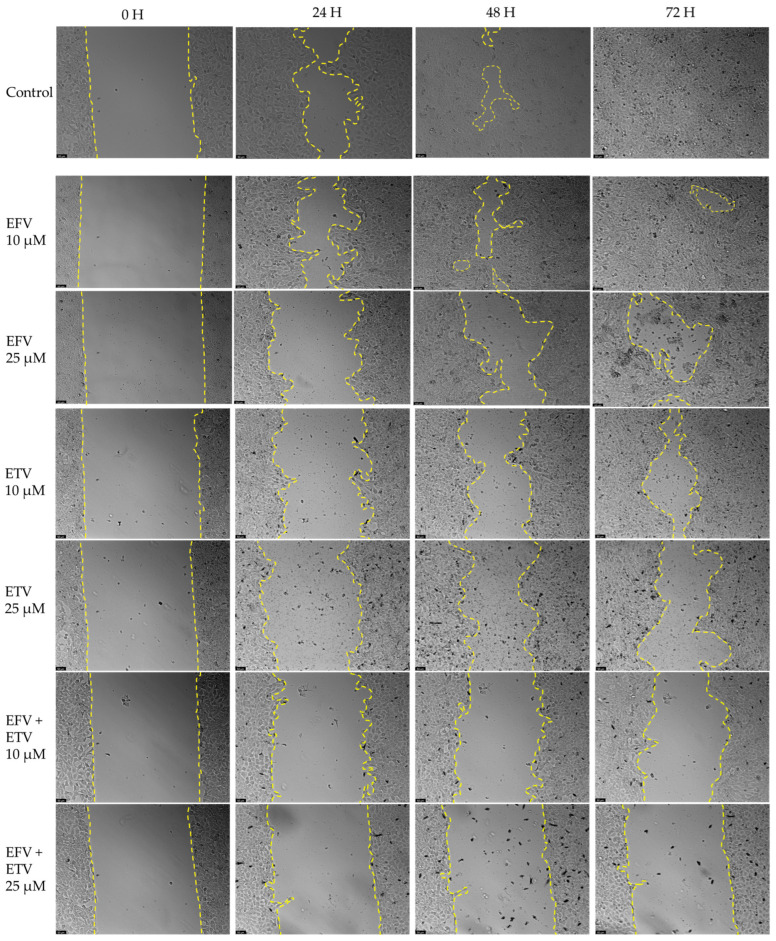
Representative images from in vitro wound healing assays in UM-UC-5 prostate cancer cells, captured at 0, 24, 48, and 72 h following treatment with EFV, ETV, or their combination. The images reflect three independent experiments performed in quadruplicate. Dashed lines are included as visual guides to indicate cell migration trends and are not intended to represent precise measurements. The scale bar represents 50 μm.

Figure 12 demonstrates the effects of efavirenz (EFV), etravirine (ETV), and their combinations on cell migration in PC-3 (prostate cancer) and UM-UC-5 (bladder cancer) cell lines over 72 h using a wound healing assay. Closure rate, indicating cell migration into the wound area, was measured at 0, 24, 48, and 72 h. Figure 13 and Figure 14 demonstrate the microscope images taken and the respective time intervals.

In PC-3 cells (Figure 12, left panel), the control group exhibited near-complete wound closure by 48 h (~95%), indicating high migratory capacity. Treatment with EFV alone (10 or 25 µM) had a minimal inhibitory effect, especially at 10 µM, where closure remained above 80%, and there was minimal to no gap already at 24 h (Figure 13). ETV alone at 25 µM significantly reduced migration, with only ~50% closure at 24 h and ~70% at 72 h. Notably, the combination of EFV + ETV showed the strongest inhibition of migration in a dose-dependent manner. The 25 µM combination resulted in only ~35% closure at 24 h and plateaued below 60% closure by 72 h, significantly lower than the control (Figure 12 and Figure 13).

In UM-UC-5 cells (Figure 12, right panel), the control group again showed robust closure (~90% at 72 h). EFV alone had a moderate inhibitory effect, particularly at 25 µM (closure rate ~65% at 72 h). ETV showed slightly greater efficacy than EFV alone, especially at 25 µM. The combination of EFV + ETV demonstrated the most pronounced reduction in migration. At the 25 µM dose, wound closure was severely impaired (~20% at 24 h and <30% at 72 h, p), with statistical significance compared to individual drug treatments throughout (Figure 12 and Figure 14).

These results suggest that ETV has, generally, a stronger effect on cancer cell motility than EFV. There was also a synergistic anti-migratory effect of EFV and ETV, especially in UM-UC-5 cell lines, particularly evident at higher concentrations, although at 10 µM of each drug, this effect is already quite evident. The combination therapy markedly impairs cell migration more effectively than either drug alone, supporting its potential utility in anti-metastatic strategies for prostate and bladder cancer.

### 2.5. Clonogenic Assays

To evaluate the capacity of EFV and ETV, alone and in combination, to interfere with the proliferation of PC-3 and UM-UC-5 cells, a clonogenic assay was performed. Cells were exposed to both drugs at 10 and 25 µM, identical concentrations to the migration assays, for 48 h.

**Figure 15:**
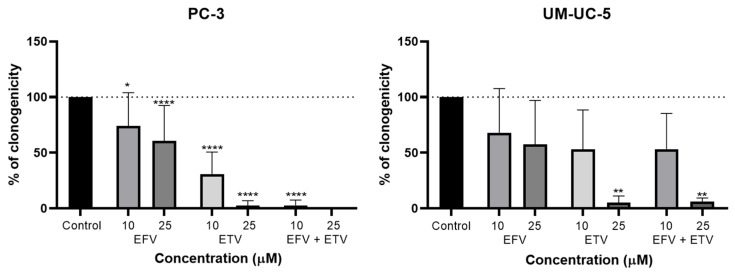
Clonogenic assay of PC-3 (**left**) and UM-UC-5 (**right**) cells treated with 10 or 25 µM EFV, ETV, and the combination of EFV + ETV at the same concentrations. Colony counts are presented as mean ± SD from three independent experiments. Statistical significance relative to the control group is indicated as follows: * *p* < 0.05; ** *p* < 0.01; **** *p* < 0.0001.

As shown in Figure 15, both EFV and ETV reduced clonogenic capacity in a dose-dependent manner, though the magnitude and statistical significance of this effect varied between cell lines. In PC-3 cells, EFV treatment led to a moderate but statistically significant reduction in colony formation at both concentrations, while ETV exerted a stronger inhibitory effect, achieving near-complete suppression of clonogenicity at 25 µM. This was also noted for the combination, but already at a concentration of 10 µM.

In UM-UC-5 cells, the overall inhibitory trend was similar, though less pronounced. While neither concentration of EFV significantly reduced colony numbers, only ETV at 25 µM led to a marked and statistically significant decrease in clonogenicity. The combination showed similar results to ETV alone, indicating that only ETV is exerting anti-proliferative effects in these cells. However, variability in response at lower doses and the relatively modest reduction in colony formation at 10 µM suggest a more selective or concentration-dependent sensitivity in this bladder cancer cell line.

These findings indicate that both EFV and ETV can impair the long-term proliferative potential of cancer cells, with ETV displaying greater potency, particularly in PC-3 cells. In these cells, the combination therapy exerts even stronger effects, resulting in the complete disappearance of colonies at 25 µM combination. The strong effect at higher concentrations highlights its therapeutic potential, although further work at lower doses may help define a safer and more selective dosing range.

### 2.6. Safety Assays—MRC-5 Cells

**Figure 16:**
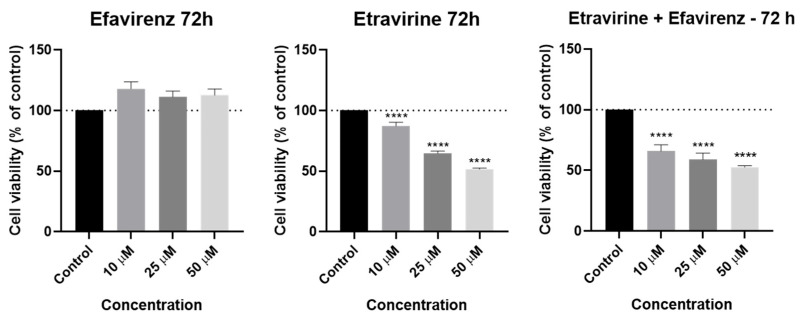
Results of MRC-5 cell viability after 72 h of exposure to EFV and ETV alone and in combination at increasing concentrations (10, 25, and 50 µM). DMSO at 0.01% was used as the vehicle. Cell viability was assessed using the MTT assay, and the results are displayed as the mean ± SEM (*n* = 3). **** Statistically significant vs. control (vehicle) at *p* < 0.0001.

**Figure 17:**
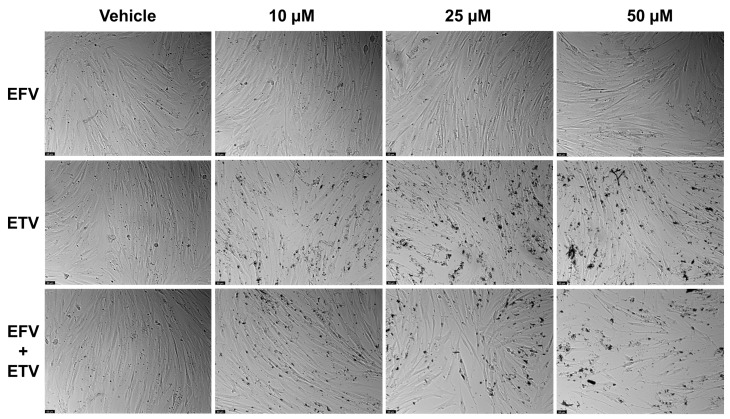
MCR-5 cell morphology was assessed following exposure to EFV and ETV alone and in combination at increasing concentrations (10, 25, and 50 µM) for 72 h (*n* = 3). Negative control cells received vehicle treatment (0.01% DMSO). The scale bar is 50 µm.

To evaluate the biosafety of these drugs, we conducted an MTT cell viability assay using human fetal lung fibroblast cells (MRC-5), for our longest time point (72 h) and various concentrations (10, 25, and 50 µM). MRC-5 cells are a widely used, well-characterized human lung fibroblast line that retains a normal diploid karyotype and shares several critical properties with primary human mesenchymal cells. They are frequently used in toxicity and biocompatibility studies to represent non-cancerous, healthy human cells, providing a robust and reproducible model for assessing off-target cytotoxicity [25,26]. The graphical results and the morphological images are represented in Figure 16 and Figure 17.

EFV is well tolerated, with no significant reduction in cell viability observed at any of the tested concentrations. In fact, slight increases in viability were observed, suggesting a minimal effect on non-cancerous fibroblasts.

ETV treatment resulted in a modest, concentration-dependent reduction in MRC-5 cell viability, with statistical significance achieved at all concentrations tested. However, it is important to note that these effects were observed following prolonged exposure (72 h), a duration that exceeds clinical plasma half-lives of both of these drugs, which are 35–50 h for EFV [27] and 30–40 h for ETV [28]. Moreover, cell viability remained above 60% even at the highest concentration (50 µM), well above the IC_50_ values obtained, suggesting partial but not severe cytotoxicity.

The combination of EFV and ETV also led to a moderate reduction in viability under the same long exposure conditions, with viability levels comparable to those seen with ETV alone. These results indicate a likely contribution from ETV as the primary driver of cytotoxicity in the combination, while EFV appears to have a minimal impact on fibroblast survival.

The morphological images from Figure 17 show that cells treated with EFV alone exhibited no appreciable morphological changes compared to the vehicle control, keeping the typical spindle-shaped fibroblast morphology across all concentrations tested. In contrast, ETV treatment induced concentration-dependent cytotoxicity, with evidence of cell rounding, detachment, and debris formation becoming prominent at 25 µM and 50 µM. Notably, co-treatment with EFV and ETV resulted in stronger morphological alterations at 25 µM and 50 µM than either drug alone, characterized by overall cell rounding, loss of cell density, and accumulation of cellular debris.

Taken together, these data suggest that EFV is well tolerated by normal human fibroblasts even at high concentrations, while ETV may induce mild to moderate cytotoxicity at elevated doses over prolonged exposure. They also indicate that for dosage, we could focus on decreasing ETV concentrations while increasing EFV’s, which could lead to a similar effect but with less pronounced cytotoxicity for healthy cells.

## 3. Discussion

Prostate and bladder cancer are both urinary tract cancers and can be related due to their proximity. Notably, bladder cancer frequently emerges as a secondary cancer after radiation therapy for prostate cancer [29]. A recent 2024 population-based study using SEER data assessed the long-term risk of secondary bladder cancer following different localized prostate cancer treatments, comparing various radiation modalities, including external beam radiation therapy (EBRT), brachytherapy (BT), combined EBRT + BT, and post-prostatectomy EBRT (RP-EBRT), to radical prostatectomy (RP) alone. The analysis included 261,609 patients treated between 2000 and 2018, with a median follow-up of 11.6 years. The study found that all radiation therapy groups were associated with a significantly elevated risk of subsequent bladder cancer compared to RP alone, with hazard ratios (HRs) ranging from 1.53 to 1.85. Notably, the risk of muscle-invasive bladder cancer and bladder cancer-specific mortality was higher among patients who received radiation, particularly those treated with EBRT, BT, or EBRT + BT (with mortality HRs up to 3.02). Bladder tumors arising post-radiation were also more likely to present with advanced-stage disease and aggressive histological features. In contrast, no significant difference was observed in the prevalence of high-grade tumors across treatment groups. These findings underscore not only an increased incidence but also a shift toward more aggressive bladder cancer phenotypes following radiation. The authors conclude that long-term surveillance for bladder cancer is warranted in prostate cancer patients undergoing radiation therapy [30].

Additionally, studies have indicated a reciprocal relationship between these two cancers, with higher incidences of prostate cancer observed in patients with bladder cancer and vice versa [31]. For instance, prostate cancer detection following radical cystoprostatectomy treatment for invasive bladder cancer has been reported at an incidence ranging from 24% to 51% [32]. Given the intimate association between prostate and bladder cancers, this study decided to research the repurposing of EFV and ETV in both.

This study demonstrates that both EFV and ETV exert significant cytotoxic effects on PC-3 and UM-UC-5 cancer cells, with varying degrees of potency, time dependence, and selectivity. ETV consistently outperformed EFV in reducing cell viability in PC-3 prostate cancer cells, showing a lower IC_50_ across all time points and inducing earlier and more pronounced cytotoxicity. EFV, while less potent in PC-3 cells, displayed more consistent and substantial cytotoxic effects in UM-UC-5 bladder cancer cells, especially with prolonged exposure.

EFV has been of great interest in drug repurposing for cancer, having shown effects in several cancers, such as triple-negative breast cancer and pancreatic cancer [23,24]. EFV at a concentration of 20 µM has been demonstrated to downregulate the proliferation of prostate cancer cells after several 96 h cycles, namely of PC-3 cells. This is caused by the alteration of nuclear functions due to DNA damage, fragmentation of nuclear lamina, and increased methylation, leading to eventual autophagy and cell death by apoptosis [33]. This validates our study, as the value used is within our range of IC_50_ obtained here.

One target that is associated with EFV and can be found both in prostate and bladder cancer is the human long-interspersed nuclear elements-1 (LINE-1) [24], which are repetitive DNA sequences composing a family of retrotransposons. LINE-1 encodes two open reading frame proteins: ORF1p, which has RNA binding characteristics, and ORF2p, which has reverse transcriptase (RT) and endonuclease (EN) functions (the latter is required for the transposition of LINE-1) [34]. LINE-1 retrostransposition is very low in healthy conditions, due to high methylation. However, LINE-1 is often found overexpressed in cancer, such as in prostate cancer, where it may be associated with initiation, progression, and poor prognosis [35], and in bladder cancer, with several epigenetically activated elements, contributing to tumor growth and genomic instability [36], making this a therapeutic target for both prostate and bladder cancer. EFV has also been demonstrated to reduce the expression of reverse transcriptase-encoding LINE-1 elements ORF1p and ORF2p, which reduces the proliferation of cancer cells due to an epigenetic alteration, marked by the reversible nature of this effect [37]. Another pathway in which EFV could have an effect is in the activation of the tumor-suppressing protein p53 by phosphorylation in the nucleus, demonstrated in glioblastoma cells with 20 and 40 µM of EFV [38]. Activation of p53 is usually associated with cell cycle arrest and apoptosis [39]. In summary, our findings align with existing literature, indicating EFV’s impact on prostate and bladder cancer treatment, likely attributed to its role in epigenetically repressing LINE-1 translation and activation of p53 tumor suppression protein.

The ETV results in UM-UC-5 bladder cancer had previously been reported, and a discussion of the possible pathways can be found in that article from our research group [22]. A potential mechanism underlying ETV’s ability to reduce cancer cell viability is the inhibition of anterior gradient protein 2 homolog (AGR2). AGR2 is a disulfide isomerase located in the endoplasmic reticulum, involved in protein folding, and has been implicated in cancer initiation, progression, and resistance to therapy [40]. It is overexpressed in both prostate and bladder cancer and associated with local tumor spread; moreover, its secretion by cancer cells makes it a potential biomarker [41,42]. It has been shown to reduce AGR2 expression in ovarian cancer, thereby suppressing cell proliferation, migration, and invasion when used alone, and inhibiting tumor growth and metastasis in vitro and in vivo when combined with paclitaxel [43]. This effect is likely mediated by AGR2′s interaction with VEGF and FGF2, which promotes angiogenesis and metastasis [44]. Notably, these results were observed at ETV concentrations of 5–10 µM across various time points [43], which, while above the IC_50_ obtained in this work, could still explain the decrease in cell viability observed in our cell lines.

For ETV, the standard therapeutic dose is 200 mg twice daily. At this dosing regimen, the mean reported steady-state C_max_ is approximately 797 ± 668 ng/mL, typically achieved within 2–4 h post-dose, which is around 1.83 ± 1.53 μM [45]. In our study, the IC_50_ values for ETV ranged from 6.74 to 14.17 µM in PC-3 cells and from 33.51 to >100 µM in UM-UC-5 cells, indicating that higher concentrations than those typically achieved in plasma are needed for effective inhibition in vitro. However, these results highlight a clear cytotoxic potential of ETV, suggesting that its anti-cancer efficacy could be enhanced through strategies such as targeted drug delivery, formulation improvements, or local tissue accumulation within tumors. For EFV, the standard clinical dose is 600 mg once daily, and the reported steady-state C_max_ is approximately 12.9 µM [46]. The IC_50_ values observed in our study ranged from 19.15 to 40.68 µM in PC-3 cells and from 24.59 to 48.32 µM in UM-UC-5 cells. Notably, the IC_50_ value in PC-3 cells at 72 h (19.15 µM) is close to, but slightly above, the average plasma concentrations attainable in patients. These findings suggest that EFV achieves cytotoxic concentrations in vitro that are near physiologically relevant plasma levels, supporting its potential for repurposing as an anti-cancer agent in specific cellular contexts.

Generally, while standard dosing of ETV and EFV may not achieve the levels reported in this study systemically, the findings open promising avenues for optimizing ETV and EFV use as repurposed therapeutics in oncology.

Combination treatment showed enhanced cytotoxicity in both cell lines, though the nature of the interaction differed. In PC-3 cells, synergy was more evident at higher concentrations and longer exposure times. In UM-UC-5 cells, however, lower concentrations of the combination already resulted in significant viability loss, implying greater sensitivity and potential for more effective combination regimens in bladder cancer. This effective combination could be because of similar effects in the cell cycle inhibition of these two drugs [38,43], where they are acting in separate pathways, which could increase the effects when compared to cells alone.

Cell migration is essential for metastasis, where cancer cells detach from the primary tumor, invade nearby tissues, and enter the bloodstream. Because of this, it is important to study whether, beyond their cytotoxic effect, our repurposed drugs have the capability of inhibiting the motility of our cancer cell lines [47]. Wound healing assays confirmed that ETV exerts stronger anti-migratory effects than EFV, with combination treatments significantly inhibiting cell motility in both lines. This is particularly noteworthy in UM-UC-5 cells, where even low-dose combinations led to marked reductions in wound closure. These data suggest that dual treatment could be a viable strategy to impair cancer cell migration and, by extension, metastatic potential.

Clonogenic assays are pivotal in cancer therapy development as they measure a cell’s ability to survive and proliferate long-term after treatment, providing critical insights into therapeutic efficacy and resistance mechanisms [48]. Clonogenic assays further supported these drugs’ repurposing, showing that both drugs suppress long-term proliferative capacity in both cell lines, especially ETV at 25 µM. Interestingly, EFV’s effects were more modest, particularly in bladder cells, where ETV again dominated in efficacy, which is similar to the migration results but contradictory to cell viability assays, where EFV had a stronger effect on bladder cancer than ETV. A combination of these two drugs showed results in both cell lines, but with a stronger effect in prostate cancer than in bladder cancer, where the effect was mainly attributed to ETV, indicating the further potential of this dual treatment, especially for prostate cancer.

The ability of ETV to significantly impair migration and clonogenicity in bladder cancer cells, particularly UM UC 5, might suggest interference with mechanisms central to the metastatic phenotype, such as epithelial-to-mesenchymal transition (EMT). Recent studies have identified prohibitin (PHB) as a key regulator of EMT and metastasis in bladder cancer through stabilization of β-catenin, thereby enhancing Wnt/β-catenin signaling. This promotes increased migration, invasion, and metastatic potential in vivo and in vitro [49]. Furthermore, a parallel study highlighted a Snai2-mediated transcriptional upregulation of NADSYN1, which binds PHB and amplifies tumor-promoting signaling. Both NADSYN1 and PHB expression were associated with poor survival outcomes [50]. These studies reinforce the importance of the PHB axis in EMT and suggest that interfering with this network could represent a novel anti-metastatic strategy. Given ETV’s significant effects on cell migration and clonogenic survival in our study, it is plausible that ETV may influence PHB-related signaling, potentially via modulation of EMT-associated transcription factors or protein interactions, warranting further pathway-targeted investigations.

The strengths of these two drugs appear to differ in UM-UC-5 cells, where EFV has a stronger immediate cytotoxic effect, while ETV ends up causing a more durable effect on proliferation and migration. These different phenotypes might be explained by variations in the drugs’ mechanisms of action. As previously mentioned, EFV has been shown to act through rapid induction of DNA damage, nuclear lamina fragmentation, and activation of p53 (via phosphorylation), which leads to relatively quick apoptotic responses in cancer cells [33,38,39]. It also downregulates LINE-1 retrotransposons ORF1p and ORF2p, helping to decrease proliferation through reversible epigenetic repression [24]. These mechanisms correspond with the stronger short-term cytotoxicity seen in viability assays. In contrast, ETV’s antitumor effects appear to emerge through slower, more durable pathways. As explained above, ETV has been shown to suppress AGR2 expression [22], a protein that promotes tumor growth and migration [40,41,43,44]. This aligns with the pronounced anti-migratory and anti-clonogenic effects of ETV seen in our current study, despite its more modest impact on short-term viability. Together, these findings suggest that EFV and ETV may operate on complementary timelines, with EFV inducing rapid cell death and ETV suppressing long-term proliferation and migration, potentially explaining the synergy observed in combination treatments.

Safety profiling in normal MRC-5 fibroblast cells showed that EFV is well tolerated at cytotoxic doses used in cancer cells. ETV, while somewhat more toxic, still maintained acceptable viability (>60%) even at 50 µM, suggesting a reasonable therapeutic index. The combination mirrored ETV’s safety profile, indicating its effect largely drives any observed toxicity. This suggests that for a treatment method, the dosages of ETV would need to be carefully examined. Perhaps compensating for lower concentrations of ETV with higher concentrations of EFV, which has been shown to be non-toxic, could be a potential approach.

Taken together, these findings provide several important directions for future research. First, mechanistic studies are needed to dissect how EFV and ETV exert their cytotoxic and anti-migratory effects, particularly regarding their influence on pathways such as p53, LINE-1, AGR2, and EMT regulators. In vivo validation of EFV and ETV efficacy using tumor models will be critical to confirm therapeutic potential and evaluate safety profiles. Additionally, optimizing drug delivery, such as through tumor-targeted formulations or by developing combination strategies, may enhance efficacy while minimizing toxicity. Finally, expanding the evaluation of EFV and ETV across diverse bladder and prostate cancer models could identify subtype-specific responses and potential biomarkers predictive of treatment sensitivity.

## 4. Materials and Methods

### 4.1. Cell Culture

EFV and ETV toxicity were assessed using the human metastatic prostate cancer cell line PC-3, the human bladder cancer cell line UM-UC-5, and the human fetal lung fibroblast cell line MRC-5. These cell lines were donated by the American Type Culture Collection (ATCC, Manassas, VA, USA), and the drugs were supplied by Sigma-Aldrich (Merck KGaA, Darmstadt, Germany). All materials were acquired from Millipore Sigma (Merck KGaA, Darmstadt, Germany), and the cells were kept in an incubator at 37 °C and 5% CO_2_ using Dulbecco’s modified Eagle’s medium (DMEM) supplemented with 10% fetal bovine serum (FBS) and 1% penicillin-streptomycin solution. Confluent cells were subcultured in fresh DMEM media with 96 h intervals of medium renewal after being trypsinized for maintenance using 0.25% trypsin-EDTA (Gibco; Thermo Fisher Scientific, Inc., Waltham, MA, USA). To conduct the experiments, 3000 (PC-3), 5000 (UM-UC-5), and 8000 (MRC-5) cells per well were seeded onto 96-well plates, and the cells were allowed to adhere overnight. Every piece of equipment used for cell culture and treatments was well-cleaned and sterilized before use. The work was carried out in a laminar flow chamber that was regularly maintained and cleaned. All materials were soaked with 70% alcohol before being placed inside the chamber.

### 4.2. Drug Treatment

EFV and ETV cytotoxicity, alone or in combination, in cancer lines was assessed using concentrations of 0.01, 0.1, 1, 10, 25, 50, and 100 μM following 24, 48, and 72 h of incubation. These concentrations span a broad range of concentrations and enable us to acquire IC_50_ values; therefore, they are the criteria we often employ in our workflow when exploring the repurposing of drugs. For the safety assays, concentrations of 10 and 25 μM were tested, with an incubation period of 72 h. The drugs were dissolved in 0.1% dimethyl sulfoxide (DMSO), which was applied to the negative control cells. Three separate studies were conducted to test each treatment.

### 4.3. Morphological Analysis

During the drug incubation period, cell morphology was observed using a Leica DMI 6000B microscope fitted with a Leica DFC350 FX camera (Leica Microsystems, Wetzlar, Germany). Image analysis was subsequently performed with Leica LAS X software (v3.7.4; Leica Microsystems, Wetzlar, Germany).

### 4.4. MTT Assay

Drug toxicity was assessed using the MTT (thiazolyl blue tetrazolium bromide) colorimetric assay. At the end of the treatment period, 100 μL of MTT solution (0.5 mg/mL in PBS; Sigma-Aldrich, Merck KGaA, Darmstadt, Germany) was added to each well. Following a two-hour incubation at 37 °C with 5% CO_2_ in the dark, the MTT solution was removed, and the resulting purple formazan crystals were dissolved in 100 μL of DMSO. Cell viability was then measured with an automated microplate reader (Tecan Infinite M200, Tecan Group Ltd., Männedorf, Switzerland) by recording absorbance at 570 nm and comparing treated samples to the negative control group.

### 4.5. Wound Healing Assay

A wound-healing assay was conducted to assess cell motility. Silicone ibidi inserts were placed in a 12-well plate to create a defined gap between cell layers. PC-3 (3 × 10^5^) and UM-UC-5 (8 × 10^5^) cells were seeded on all sides of the insert in 110 µL per side and allowed to adhere for 24 h. After insert removal, four consistent gaps (“wounds”) were formed. Wells were rinsed twice with PBS to remove non-adherent cells, then treated with EFV and ETV, alone or in combination (10 and 25 µM), for 48 h under standard conditions (37 °C, 95% humidity, 5% CO_2_). Wound areas were imaged at 0, 24, 48, and 72 h using 100× magnification. The percentage of wound closure was quantified using ImageJ (FIJI, version 1.53, NIH, Bethesda, MD, USA) by comparing the remaining gap at each time point to the initial wound area (0 h).

### 4.6. Clonogenic Assay

PC-3 and UM-UC-5 cells were seeded at a density of 100 cells per well in standard 6-well plates, with three technical replicates per condition. Once cells reached approximately 80% confluence, they were incubated overnight and subsequently treated with or without EFV and ETV (10 and 25 µM, alone and in combination) for 48 h. Following drug exposure, the medium was replaced with fresh, drug-free complete medium, and cultures were maintained for an additional 12 days at 37 °C in a humidified atmosphere containing 5% CO_2_. The medium was refreshed every 2 days throughout the incubation period. After 14 days, colonies were fixed and stained using a 0.5% (*v*/*v*) crystal violet solution. Colonies were counted manually and were included in the analysis if they were non-overlapping, clearly visible, and greater than 0.5 mm in diameter.

### 4.7. Statistical Analysis

All data are presented as mean ± SEM, and graphs were generated using GraphPad Prism 9 (GraphPad Software Inc., San Diego, CA, USA). Statistical analysis between experimental drug groups and the negative control was performed using one-way ANOVA followed by Dunnett’s multiple comparisons test. For comparison of combination treatments with individual drug effects at corresponding concentrations, a two-way ANOVA was applied. A significance threshold of *p* < 0.05 was used. Viability data were normalized to the negative control, and dose–response curves were generated by plotting logarithmic drug concentrations using non-linear regression to calculate IC_50_ values.

## 5. Conclusions

This study highlights the potential of the antiretroviral drugs efavirenz (EFV) and etravirine (ETV) as repurposed anti-cancer agents against prostate and bladder cancer cell lines. Both compounds demonstrated notable cytotoxicity in a time- and concentration-dependent manner, with ETV showing greater potency in PC-3 cells and EFV exhibiting more consistent effects in UM-UC-5 cells. Combination treatments revealed additive or synergistic effects, particularly in bladder cancer cells, enhancing cytotoxicity and impairing cell migration more effectively than either agent alone. Importantly, EFV showed a favorable biosafety profile in non-cancerous MRC-5 fibroblasts, while ETV induced only moderate cytotoxicity at higher concentrations and longer exposures. These results suggest that EFV and ETV hold promise as selective therapeutic options for urinary tract cancers, warranting further investigation in in vivo models and mechanistic studies to validate their clinical relevance and optimize dosing strategies.

## Figures and Tables

**Table 1:** Summarized values of IC_50_ of EFV and ETV in both cell lines and all time points.

Time (h)	PC-3 IC_50_ (µM)		UM-UC-5 IC_50_ (µM)	
	Efavirenz	Etravirine	Efavirenz	Etravirine
24 h	40.68	14.17	48.32	93.18
48 h	23.40	8.22	35.68	>100
72 h	19.15	6.74	24.59	33.51

## Data Availability

Data are contained within the article.
